# Seizures and Postictal Psychosis in a Patient With Retrocerebellar Arachnoid Cyst: A Case Report

**DOI:** 10.7759/cureus.24935

**Published:** 2022-05-12

**Authors:** Rittika Biswas, Ishan Sen

**Affiliations:** 1 General Medicine, Calcutta National Medical College and Hospital, Kolkata, IND; 2 Geriatrics, University Hospitals Dorset, Poole, GBR

**Keywords:** intracranial, retrocerebellar, seizure, arachnoid cyst, postictal psychosis

## Abstract

Retrocerebellar arachnoid cysts are uncommon intracranial tumors, especially rare in adults. Although asymptomatic in the majority of cases, they may cause a variety of symptoms including convulsions. The causal involvement of these cysts in postictal psychosis, however, remains a topic that is not well-explained in the literature.

An 85-year-old Asian man presented with recurrent episodes of convulsions for the last seven months along with preceding headaches and postictal psychotic symptoms. MRI of the head revealed a retrocerebellar arachnoid cyst. He was commenced on symptomatic pharmacological therapy after he refused surgical intervention and remained symptom-free till his discharge from the hospital before being eventually lost to follow-up.

This case report focuses on the rare occurrence of a retrocerebellar arachnoid cyst with seizures and postictal psychosis and illustrates the necessity of further case studies and research to identify and explore the potential causal relationship between arachnoid cysts and psychosis.

## Introduction

Intracranial arachnoid cysts are a rare occurrence in adults with a prevalence of 0.2-1.4% in the general population [1]. These cysts are congenital malformations, lined by arachnoid epithelium and filled with cerebrospinal fluid [2]. They are usually unilateral and more commonly occur in the middle cranial fossa. Males have a stronger predilection of having temporal fossa cysts, while females are more likely to have them in the cerebellopontine angle (CPA) [3].

Intracranial arachnoid cysts are largely asymptomatic, especially those located in the posterior cranial fossa. However, rarely, the posterior cranial fossa arachnoid cysts may give rise to symptoms like dizziness, headache, ataxia, hearing loss, and convulsive episodes [4].

There has been some discussion regarding the relationship between psychosis and arachnoid cysts, but while some patients with arachnoid cysts have manifested psychotic symptoms, there is still insufficient evidence to establish that the psychosis was secondary to the cyst in those cases [5].

Postictal psychosis most commonly occurs in patients with chronic epilepsy or those with an underlying psychiatric disorder. They are also seen in patients with a history of head trauma, widespread CNS injury, borderline intelligence, and EEG slowing [6].

Here, we report a unique case of retrocerebellar arachnoid cyst presenting with active convulsions with a brief history of postictal psychosis.

## Case presentation

The authors report an 85-year-old male admitted with several episodes of generalized tonic-clonic seizures for the last seven months. The seizures were always preceded by a headache beginning one to two hours before. The patient also reported experiencing postictal symptoms like transient blurring of vision, loss of recent memory, behavioral changes, and disorientation to time, place, and person. The symptoms usually progressed for one to two days, before gradually improving over two to three weeks, following which the patient returned to his baseline (a Mini-Mental State Examination (MMSE) score of 26/30).

Collateral history from the primary carer of the patient revealed psychotic episodes, which usually began 12-48 hours after seizures and lasted for two to three weeks. The patient would then slowly regain cognitive function, and attain baseline behavior. He would remain stable till the next bout of convulsions a few months later followed by postictal psychosis. The psychotic episodes followed a distinct pattern where it would usually begin with mild symptoms like memory loss and disorientation to time, place, and person, and would quickly progress to full-blown aggression, paranoia, and agitation over one to two days. The patient would be unable to recognize his family members and carry out basic motor activities like eating, and would not respond to any instructions. He would be verbally aggressive and physically violent. These symptoms would gradually abate over the covering two to three weeks and the patient would return to his baseline cognition.

The patient also complained of recurrent headaches for the last two years, which were occasionally accompanied by bouts of projectile vomiting without preceding nausea. These episodes of headache would occur every two to three months but started becoming more frequent in the last few months.

He also reported vertigo and tinnitus for the last year with difficulty maintaining balance and a tendency to fall on one side. Over the year, his symptoms progressed to include uncoordinated speech and an inability to shave and bring food to his mouth. The patient did not have any history of hearing loss.

There was no history of abnormal taste or smell, difficulty in chewing, tingling or numbness on one side of the body, limb weakness, epiphora, facial asymmetry, vocal changes, or dysphagia. No history of any symptoms of stroke or cord compressions like paraesthesia, girdle-like sensation, or root pain was present. There was no history of tremors. There was also no history of urinary incontinence, fecal retention, or abnormal sweating. The patient did not complain of fever, neck pain, photophobia, or phonophobia. He did not report any history of head trauma, meningitis, or tuberculosis. No recent travel history was present either.

There was no significant background medical history including no history of epilepsy since childhood or any developmental anomaly. No family history of related medical issues was present including no history of kidney disorders, tuberculosis, brain tumors, or epilepsy. The patient was not on any regular medication. There was no history of addiction to alcohol, smoking, or drugs.

On immediate post-ictal examination, the patient was conscious, but not oriented to time, place, or person, and was able to follow only simple instructions and commands. Bilateral plantar responses were extensor. The patient did not have an ataxic gait, at present, and Romberg's test was negative. The patient had a generalized lack of coordination in bilateral upper and lower limbs, as he was unable to perform the finger-nose and heel-to-shin tests successfully, which led us to suspect a cerebellar lesion. There was no abnormality noted on the examination of cranial nerves. Ocular examination was within normal limits and nystagmus was absent. Vitals including blood pressure and capillary blood glucose level were within normal limits.

**Figure 1:**
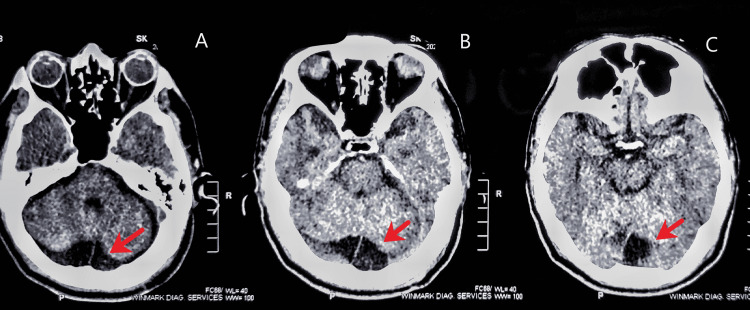
Axial sections of head CT showing retrocerebellar hypodense lesion

An urgent CT scan of the head was done, which revealed a hypodense lesion behind the cerebellum along with cortical atrophy (Figure 1). Routine laboratory investigations including full blood count, urea and electrolytes, lipid profile, liver function, and thyroid function tests were all within normal limits. A urine sample was sent for microscopy and culture, which revealed no growth of any organism. Chest X-ray, ECG, and arterial blood gas (ABG) were within normal limits. An EEG performed following stabilization of seizures was found to be normal.

**Figure 2:**
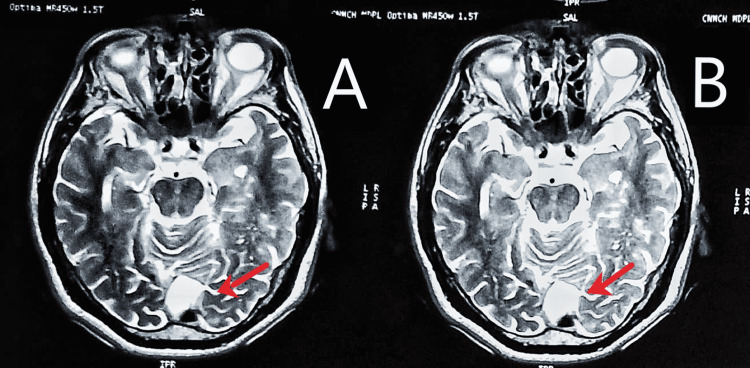
Head MRI (T2-weighted) in the axial plane

**Figure 3:**
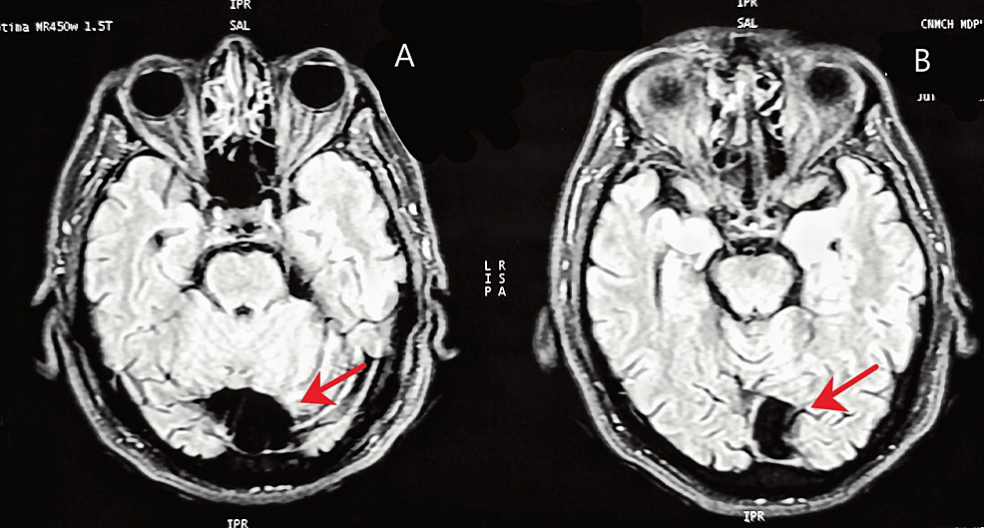
Axial sections of head MRI (T1-weighted) showing retrocerebellar arachnoid cyst

**Figure 4:**
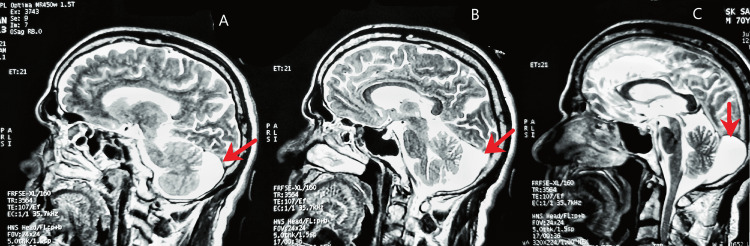
Head MRI (T2-weighted) in the sagittal plane

An MRI scan of the head was done, which showed an arachnoid cyst in the retrocerebellar area of the posterior fossa, along with multifocal tiny ischemic gliosis in peri and periventricular white matter and centrum semiovale on both sides in a background of diffuse cerebral and cerebellar atrophy (Figures 2-4). The small vessel ischemic changes and the cortical atrophy were consistent with probable age-related changes.

Interestingly, an ultrasound scan of the whole abdomen revealed an incidental solitary cyst, measuring 30 x 24 mm in the VII segment of the liver. Since it was asymptomatic and not large, we decided against intervention and planned for an ultrasound follow-up.

When the patient was initially admitted with active convulsions, he was treated according to hospital protocol with a 4 mg bolus of lorazepam intravenously. However, the seizures did not cease at 10 minutes, and another 4 mg bolus of intravenous lorazepam was administered. Once that failed to terminate the seizures, a loading dose of 1000 mg of levetiracetam was administered intravenously, which finally stopped the convulsions. The patient was then put on intravenous levetiracetam 500 mg twice a day, which was switched to oral tablets five days later, once the patient had improved. A neurosurgery referral was sent for consideration of decompression of the symptomatic cyst. Following an extensive discussion with the neurosurgery team, the patient and his next-of-kin refused to consent to the procedure and explained their wishes of continuing medical treatment. He remained seizure-free for the remainder of the hospital stay and was discharged once clinically stable on oral levetiracetam 500 mg 12 hourly. He was booked for outpatient follow-ups in the neurosurgery, medicine, and psychiatry clinics, but was lost to follow-up.

## Discussion

Arachnoid cysts are rare congenital tumors that result from a collection of cerebrospinal fluid (CSF) within the arachnoid membrane and are mostly seen in the pediatric age group [1,2]. Arachnoid cysts in the posterior fossa are uncommon, with only 5-10% of arachnoid cysts being located there. In the posterior cranial fossa, arachnoid cysts are usually found in the cerebellopontine angle, cerebellum, and fourth ventricle [4].

Most arachnoid cysts are diagnosed incidentally with the majority of the patients being asymptomatic. However, when clinical symptoms are present, the most frequent manifestations are headache, dizziness, and convulsions [7]. It is relevant to note here that the location of the lesion often determines the type of signs and symptoms experienced by the patient. In a study of 45 arachnoid cyst patients, Mazurkiewicz-Bełdzińska et al. found a strong correlation between patients presenting with epilepsy and left temporal arachnoid cysts [8].

Posterior fossa cysts typically pose a diagnostic challenge, as they tend to present with a wide array of non-specific symptoms such as headache, dizziness, hearing loss, tinnitus, lower cranial nerve palsies, cerebellar signs, pyramidal signs, psychomotor retardation, and seizures [4]. However, epileptiform activity associated with posterior fossa arachnoid cysts is an extremely rare occurrence [9].

Our patient presented with recurrent episodes of headache, cerebellar signs, and generalized tonic-clonic seizures, followed by postictal psychosis. While cerebellar signs and symptoms can be attributed to the retrocerebellar location of the arachnoid cyst, they do not explicitly explain the cause of seizures or psychosis in the absence of visible evidence of increased intracranial pressure (such as hydrocephalus on CT).

Interestingly, in a case described by Bahk et al., the cyst compressed the left temporal lobe and surrounding areas that are known to be both epileptogenic and psychogenic [10]. In our patient, the cyst was retrocerebellar without any obvious evidence of increased intracranial pressure on CT and MRI, thereby suggesting an alternate cause of seizures. Recent studies have shown the likelihood of cerebellar inhibition on cortical epileptiform activity. Although the pathophysiology is not yet described, cerebellar lesions have been theorized to cause seizures owing to the disinhibition of cortical areas or intrinsic epileptic activity of cerebellar lesions [11]. This would explain the seizures in our patient with a posterior fossa cyst.

Postictal psychosis is a well-known complication of chronic epilepsy that usually follows a cluster of tonic-clonic seizures. The postictal confusion state is followed by a characteristic lucid interval, which is then followed by the psychosis that may last from days to weeks. A certain degree of delirium and confusion has often been found to coexist with the more overt psychotic symptoms like thought and mood disorder, affective changes, aggression, and audiovisual hallucinations [6].

Interestingly, Vakis et al. described intermittent rises in intracranial pressure in a case of atypical psychosis with an intracerebral arachnoid cyst. Despite neuroimaging studies failing to detect these intermittent rises, the fact that the patient’s symptoms improved following the placement of a permanent shunt led the authors to consider an association between intracranial pressure changes and psychosis [12].

Our patient reported clear episodic behavioral changes manifesting as aggressive symptoms that align with the definition of psychosis. The fact that these symptoms would typically progress for the first one to two days before resolving completely within two to three weeks led us to consider the association between psychotic manifestations and episodes of increased intracranial pressure. Similar to the case described by Vakis et al., neuroimaging failed to detect any changes in our patient.

In a unique case of an arachnoid cyst in the trigone of the right lateral ventricle, Wong et al. described short psychotic episodes in a young female one to two hours after she had been recumbent in bed, possibly resulting from the blockade of the temporal horn by the cyst leading to local ischemia. The authors believed that the characteristic psychosis in their patient was a form of a partial complex psychomotor seizure [13].

Non-convulsive status epilepticus (NCSE) was a differential diagnosis that was considered. However, as the patient has a history of returning to his baseline cognitive functioning after the seizures, and has no history of subtle motor features (eye blinking, twitching) during episodes of altered sensorium, this possibility was considered less likely [14].

Even then, the association of psychosis with intracranial arachnoid cyst remains scarce in literature [5]. In their case report, Bahk et al. described a brief psychotic episode in a patient with an arachnoid cyst and explored the causal relationship between the two [12]. Similar to that case, our patient had no past or familial history of psychiatric or seizure disorders, developed psychosis following seizures only for the last seven months, and had clear neurological symptoms of the arachnoid cyst for the same duration, suggesting that the psychosis was secondary to the arachnoid cyst. There were no other relevant findings on laboratory investigations that would explain another cause. Considering the inadequate evidence base in current literature, the association of intracerebral arachnoid cysts with episodic psychosis needs to be studied in further detail.

Furthermore, impairment of cognitive functions including memory, verbal perception, and finer executive functions have been associated with arachnoid cysts [1,15]. This is consistent with the decline of higher mental functions in our patient. These changes, however, are reversible in nature, with decompressive procedures leading to a definite improvement [1].

Surgical intervention aimed at decompressing the lesion is usually the preferred and effective approach while dealing with symptomatic posterior fossa arachnoid cysts. Evidence shows that decompression techniques have higher efficacy than conservative methods in reducing symptoms [16]. However, in patients presenting with neuropsychiatric features, targeted symptomatic treatment in the absence of surgery has been shown to relatively improve psychiatric symptoms including psychosis [17].

Our patient was initially treated with anti-epileptics to control the seizures and was referred to the neurosurgery department for consideration of a decompressive procedure. He, however, refused to undergo surgery and opted to remain under pharmacological treatment in the medical ward till his discharge.

## Conclusions

In conclusion, this case highlights the rare occurrence of symptomatic retrocerebellar arachnoid cyst along with seizures and postictal psychosis. The report emphasizes the importance of exploring a possible causal relationship between intracranial arachnoid cysts and postictal psychosis and provides a platform for further descriptive studies and research into the pathomechanism behind it. Additionally, the role of the cerebellum in cortical disinhibition and its intrinsic epileptic activity needs to be investigated further.
